# Label-free microcargo delivery and controllable release in complex fluidic environments based on autonomous magnetic microswarm

**DOI:** 10.1038/s41378-026-01392-0

**Published:** 2026-07-24

**Authors:** Zhixin Wu, Liushuai Zheng, Lei Fan, Tanyong Wei, Jinyang Gao, Xue Qi, Lei Zhang, Huanting Shi, Bingdan Wang, Renkai Guo, Hao Yan, Qiulin Tan

**Affiliations:** 1 State key Laboratory of Extreme Environment Optoelectronic Dynamic Measurement Technology and Instrument, North University of China, Taiyuan, 030051 China; 2 Key Laboratory of Micro/nano Devices and Systems, Ministry of Education, North University of China, Taiyuan, 030051 China; 3 School of Mechanical Engineering, Suzhou University of Science and Technology, Suzhou, 215000 China; 4 School of Mechanical Engineering, Hubei University of Technology, Wuhan, 430068 China; 5 Department of Biliary and Pancreatic Surgery, First Hospital of Shanxi Medical University, Taiyuan, 030001 China

**Keywords:** Microfluidics, Electrical and electronic engineering

## Abstract

Magnetic microswarms exhibit great potential for targeted delivery because of their excellent controllability and environmental adaptability. Enabling label-free cargo delivery and controllable release while endowing microswarms with resistance to environmental disturbances is key to expanding their application scope. In this study, we present a label-free microcargo delivery strategy based on a magnetic microswarm actuated by a magnetic tweezers system. In this approach, high-frequency magnetic fields enable autonomous cargo capture, while low-frequency fields trigger controlled release, forming a simple yet effective frequency-switching mechanism. Owing to the high-intensity magnetic field produced by the magnetic tweezers system, the microswarm exhibits significantly improved anti-interference capability, ensuring stable transport even under dynamic flow conditions. Leveraging visual feedback, the microswarm autonomously captures and stably transports various microscale cargos, including polystyrene microspheres and cell spheroids up to 400 µm in diameter. In complex structures and flowing fluid environments, microswarms that carry cargo successfully resist fluid impacts and achieve autonomous navigation. Furthermore, the controllable release of nonmagnetic cargos is realized through a frequency-switching mechanism. When a microswarm carries multiple cargos, small cargos are usually discharged first, indicating the potential of this release mechanism to sequentially release multiple cargos. This strategy circumvents the risks associated with permanent magnetic labeling and enhances the microswarm’s anti-interference capability, providing essential technical support for the practical translation and clinical application of magnetic microrobots.

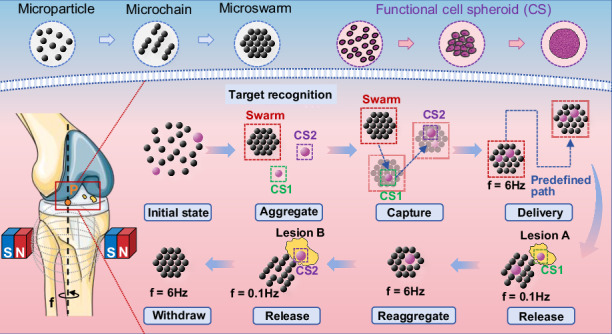

## Introduction

Microrobotics is a rapidly developing interdisciplinary field integrating materials science, robotics, and biomedical engineering^1,2^. Owing to their small size and high maneuverability, microrobots can access narrow and complex anatomical regions that are often inaccessible using conventional minimally invasive devices^3,4^. Consequently, microrobotics is spurring considerable interest in the precise delivery of microcargos, such as pharmaceuticals, stem cells, and diagnostic agents^5,6^. Actuated by external fields, including magnetic, ultrasound, optical, or electric fields, microrobots can navigate precisely and deliver microcargos to designated sites, thereby significantly enhancing therapeutic precision and efficacy^7–10^. Among the aforementioned external fields, magnetic fields provide precise, control and directional actuation with excellent biocompatibility and deep tissue penetration, making them particularly well suited for in vivo biomedical interventions^11,12^. Magnetic microrobots that can successfully perform drug delivery and targeted cell transport have recently been demonstrated, highlighting their strong clinical potential^13–16^.

However, individual magnetic microrobots are constrained by their limited payload capacity, and in vivo imaging technologies often possess insufficient resolution for real-time tracking at the microscale^17–19^. Magnetic microswarms comprising multiple microrobots resolve this problem by enhancing the load capacity, improving imaging contrast, and displaying reconfigurability, allowing adaptation to complex and dynamic biological environments^20–22^. Microswarms actuated by dynamic magnetic fields have been employed for nanoparticle delivery^23–25^. Current swarm-based manipulation of nonmagnetic targets usually requires magnetic labeling or the fabrication of composite magnetic carriers^26–32^, which may alter the biological functions of sensitive payloads such as cell spheroids, proteins, and drug molecules^33–35^. Furthermore, residual magnetic components are difficult to retrieve after delivery, posing potential biotoxicity risks and limiting clinical safety^36,37^. Therefore, a non-invasive strategy for manipulating nonmagnetic targets without magnetic modification is required. Several indirect approaches for controlling cargo have recently been explored^38–40^. A paramagnetic multimodal microswarm has been utilized as an end effector to automatically manipulate micro-objects, which significantly enhances the degree of automation of the system^41^. Furthermore, a novel planning strategy further improves the autonomy of magnetic microrobotic systems for micromanipulation tasks^42^. Despite these successful demonstrations, the anti-interference capabilities of the delivery process require further improvement^43–45^.

In this paper, we propose a label-free strategy for microcargo delivery using a microswarm composed of Fe_3_O_4_ nanoparticles. Under the control of a magnetic tweezers system, the microswarm enables capture, delivery, and release of microcargo. Integrated with a recognition algorithm, the microswarm achieves automated sequential capture based on the cargo size. Moreover, the cargo-carrying microswarm demonstrates autonomous navigation within complex structures. The high-intensity magnetic field generated by the magnetic tweezers system further enhances the microswarm’s anti-interference capability and cargo delivery performance. Under dynamic flow conditions, the microswarm successfully delivers cargo both downstream and upstream. Furthermore, we introduce a controllable release mechanism based on frequency switching. Tuning the rotation frequency dynamically adjusts the swarm morphology to independently release multiple cargos. During the release of cargos with different sizes, smaller targets are typically released first, indicating the potential application of this release mechanism for sequential multicargo release. This strategy avoids the safety hazards associated with permanent magnetic labeling and enhances the stability of the delivery process, exhibiting strong potential in intra-tissue targeted therapy.

## Results

### A label-free microcargo delivery mechanism based on magnetic tweezers actuated microswarms

**Fig. 1:**
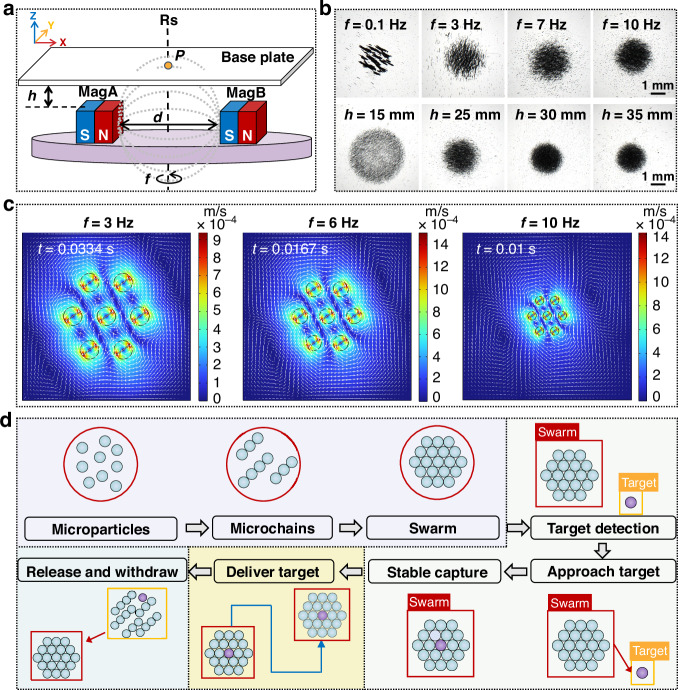
Overview of the microswarm-based cargo delivery system and operational workflow. **a** Schematic of the magnetic tweezers operating principle. **b** Morphological evolution of the microswarm under varying rotation frequencies (f = 0.1–10 Hz, h = 20 mm) and field height (h = 15–35 mm, f = 6 Hz). **c** Simulated flow fields around microswarms in three representative configurations: loose structure at f = 3 Hz (t = 0 s and t = 0.01 s), moderate structure at f = 6 Hz (t = 0 s and t = 0.0167 s), and dense aggregate at f = 10 Hz (t = 0 s and t = 0.0334 s). The white arrows indicate the local flow velocity direction. **d** Workflow of the magnetic microswarm-mediated cargo delivery

In this study, a custom-built magnetic tweezers system was used to manipulate a magnetic microswarm^46^. The magnetic tweezers system comprised an imaging device, a host computer, a base plate, permanent magnets, a rotating disk, and a translation stage (Fig. S1), and its operating principle is illustrated in Fig. 1a. A controllable rotating magnetic field was generated by a pair of rectangular permanent magnets (10 mm × 10 mm × 10 mm) with a polar distance (d) of 15 mm. The spatial distribution of the magnetic field was simulated using COMSOL Multiphysics, as shown in Fig. S2. The magnets increase the strength of the magnetic field, improving the anti-interference capabilities of the microswarm. Under the influence of the magnetic field, microparticles eventually assemble into a microswarm at point P, defined as the intersection of the rotating shaft (Rs) of the disk and the base plate. When the position of P changes, the microswarm moves with it. Thus, this characteristic was used to achieve the automated navigation of the microswarm.

The morphology of the magnetic microswarm is determined by the magnetic field height (h) from the magnets to the base plate, and the rotation frequency (f) of the magnets. In this study, Fe_3_O_4_ magnetic microparticles were used to form the magnetic microswarm. Figure 1b shows the morphological evolution of the microswarm under different magnetic field rotation frequencies (0.1–10 Hz) at a height of 20 mm and at different heights (15–35 mm) at a frequency of 6 Hz. The manipulation of microcargos can be achieved by modulating the morphology of the magnetic microswarm. When f < 1 Hz, the microswarm maintains a long chain-like configuration with large internal pores, which facilitates the release of the captured cargo. When f exceeds 1.5 Hz, the enhanced hydrodynamic force breaks the long chains, and the microswarms gradually reorganize into denser aggregates with clear outlines, thereby enabling the efficient capture of cargo. In addition to f, the parameter h significantly affects the microswarm morphology. As h increases, the microchains shorten, and the microswarm becomes more compact.

This structural evolution significantly affects the hydrodynamic behavior in its vicinity, thereby modulating the performance of the microswarm in target manipulation. A finite element model was established in this study to gain an in-depth insight into the intrinsic correlation between the microswarm structure and flow field characteristics. The microchain structures were simplified to rectangular geometries^47^. The model simulated the flow field distribution around the microswarm at three representative frequencies (Fig. 1c). The rotational motion of the microswarm induced a vortex-like flow field centered on its core. As the microswarm approached the cargo, the resulting pressure gradient drew the cargo to the edge of the microswarm and guided its inward spiraling motion toward the center, thereby enabling efficient encapsulation (Fig. S3). During directed locomotion, the encapsulated cargo was stably retained within the microswarm, facilitating synchronized transport.

The flow field distribution generated by the microswarm was closely correlated with the microchain scale (Fig. S3). At a low frequency (f = 3 Hz), the microchains were excessively elongated, and the microswarm structure was loose, leading to reduced internal fluid velocity, weak pressure gradients, and ultimately low cargo capture efficiency. As the frequency increased (f = 6 Hz), the microchains reached a moderate length with reduced spacing, leading to a higher fluid velocity that enhanced the capability of the microswarm for cargo capture and retention. However, when the frequency was excessively high (f = 10 Hz), the microchains became too short and densely packed and formed a compact aggregate that restricted the internal fluid motion and reduced the cargo transport efficiency. Therefore, an optimal frequency threshold exists at which the microswarm transport performance is maximized, providing a theoretical foundation for subsequent precise manipulation.

Furthermore, we propose a frequency-switching strategy to release the captured cargo. When f was reduced below 1 Hz, the microchains rapidly restructured and elongated, forming a thick rod-shaped configuration. In this state, the microswarm exhibits a loose architecture with a significantly weakened flow-field capture force. We hypothesize that the rotational motion of this rod-shaped structure generates a thrust on the internal cargo, driving its migration toward the microswarm edge and causing it to gradually separate, thereby achieving controlled cargo release.

Figure 1d illustrates the complete workflow for delivering cargo using a microswarm. The magnetic microparticles injected into the working area are typically dispersed. Upon the application of a rotating magnetic field, they self-assemble into a microswarm. The visual feedback system tracks the real-time positions of the microswarm and cargo, enabling autonomous capture. After stable encapsulation, the microswarm carries the cargo for directional delivery. A frequency-switching mechanism is triggered upon reaching the release area. The subsequent restoration of the high-frequency magnetic field rapidly reconfigures the microswarm into a dense aggregate and withdraws it from the release area. This strategy enables label-free cargo delivery and controllable release. Moreover, the application of a high-strength magnetic field endows the microswarm with enhanced resistance to environmental disturbances, offering a practical technical pathway for minimally invasive delivery applications in complex biological environments.

### Microswarm motion control and cargo-carrying performance

**Fig. 2:**
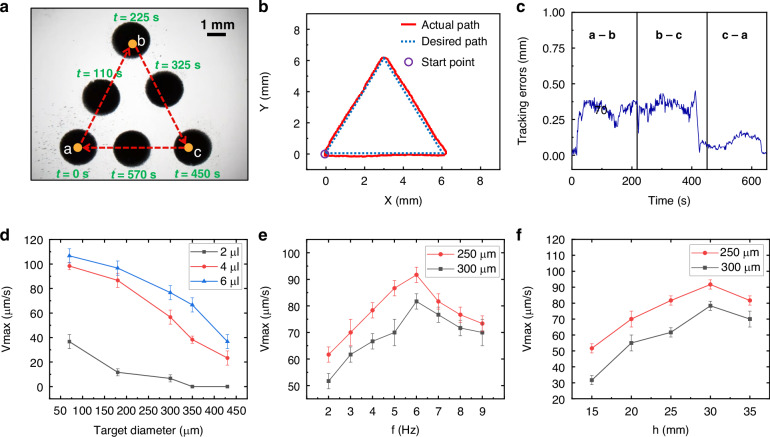
Trajectory tracking and delivery performance characterization of magnetic microswarms. **a** Microswarm navigation along a predefined triangular trajectory. **b** Superimposed trajectories of the actual (red solid line) and desired (blue dotted line) paths. **c** Tracking errors between the actual and target trajectories. **d** *V*_max_ of microswarm composed of 2, 4, and 6 µL of magnetic particles during the transport of PS (diameter: 70–430 µm). **e** *V*_max_ of PS-carrying microswarm for varying f, where h is maintained at 30 mm. **f** *V*_max_ of the PS-carrying microswarm for varying h, maintaining f at 6 Hz. The error bars represent the standard deviation calculated from three repeated experiments

The motion performance of the microswarm was evaluated through directional movement experiments (Supplementary Movie 1). Figure 2a shows the microswarm moving along a triangular trajectory under the guidance of the magnetic tweezers. Figure 2b shows the superimposed trajectories of the actual path (red solid line) and desired path (blue dotted line) of the microswarm. Figure 2c shows the tracking errors between the actual and desired path. Despite the sharp turn of 60° during the experiment, the microswarm stably followed the desired path, with the maximum tracking error remaining within 0.5 mm. This excellent tracking capability demonstrates the potential of the system for microscale-targeted delivery.

The cargo-carrying performance was systematically evaluated for microswarms with varying doses of Fe_3_O_4_ particles (Fig. 2d). Experiments were conducted under a magnetic field with f = 6 Hz and h = 30 mm, using polystyrene microspheres (PSs) as cargos with diameters of 70–430 µm. As the particle dose increased, the maximum velocity (*V*_max_) of the cargo-carrying microswarm increased. However, the hydrodynamic drag force to be overcome by the microswarm increased significantly with the cargo diameter, which progressively reduced *V*_max_. When the cargo exceeded the structural bearing limit of the microswarm, it failed to follow the movement of the microswarm. Experiments demonstrated that microswarms formed from 2, 4, and 6 µL of magnetic particles stably transported PSs with diameters of 70–430 µm. Owing to its performance advantages, the microswarm assembled with 6 µL of particles was used in subsequent experiments.

In addition to the particle dose, both f and h influence the cargo-carrying capacity of the microswarm. The *V*_max_ of microswarms transporting 250 and 300 µm PSs under different f and h is shown in Fig. 2e, f. At a fixed h of 30 mm, *V*_max_ increases gradually with f, reaching a peak at 6 Hz. Further increase in f leads to denser microswarm structures, resulting in reduced transport efficiency. Similarly, when f is maintained at 6 Hz, increasing h initially promotes structural compaction and enhances *V*_max_, which peaks at 30 mm before decreasing with a further increase in h. These experimental results are consistent with the simulation predictions, indicating that f and h jointly govern the structural morphology and hydrodynamic characteristics of the microswarms, thereby modulating their delivery capabilities. Based on this optimization, the parameter set (f = 6 Hz, h = 30 mm) was adopted in subsequent experiments to achieve maximal cargo velocity and efficient delivery.

### Autonomous capture and controllable release of microcargos

**Fig. 3:**
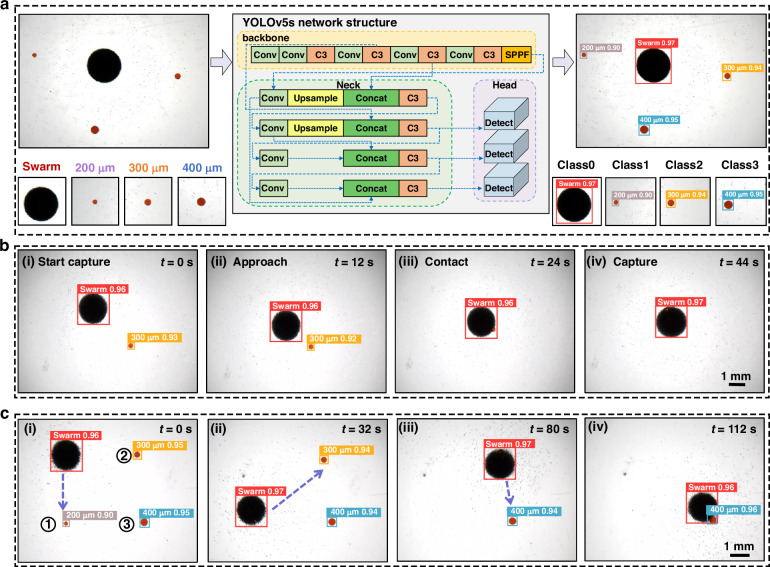
Target recognition and autonomous capture of microcargo. **a** Visual comparison of raw images and YOLOv5s detection results. **b** Autonomous capture of a single PS by a microswarm, guided by the magnetic tweezers with YOLOv5s-based visual feedback. **c** Programmable capture of multiple PSs by the microswarm in ascending order of size

By integrating a deep learning algorithm, the system can autonomously locate the microswarm and cargo, enabling autonomous capture and significantly improving automation in micromanipulation. In this study, we employed the YOLOv5s deep learning model for real-time visual perception^48,49^. Nonmagnetic cargo was represented by red PSs with diameters ranging from 200 to 400 μm. A representative training dataset was built from images of PSs and the microswarm, acquired under diverse spatial configurations. The YOLOv5s model was trained using this dataset, enabling the detection of microswarm and multiscale cargo in complex backgrounds (Fig. 3a).

As shown in Fig. 3b, the microswarm autonomously captured a single PS with a diameter of 300 µm. As the microswarm approached, the PS was first entrained by the vortex flow field at its periphery, gradually drawn into its interior, and finally stably encapsulated (Supplementary Movie 2). Figure 3c and Supplementary Movie 3 further demonstrate the microswarm’s size-selective capture of PSs with diameters of 200, 300, and 400 µm, highlighting its robust multitarget discrimination and programmable manipulation capabilities. However, when targets are densely clustered, the capture and transport of one microbead may inevitably disturb the positions and states of adjacent beads, which imposes a practical limitation on the selectivity performance within dense multitarget clusters. Further optimization of the control strategy is required in future work to improve the selectivity of the microswarm in multitarget manipulation.

**Fig. 4:**
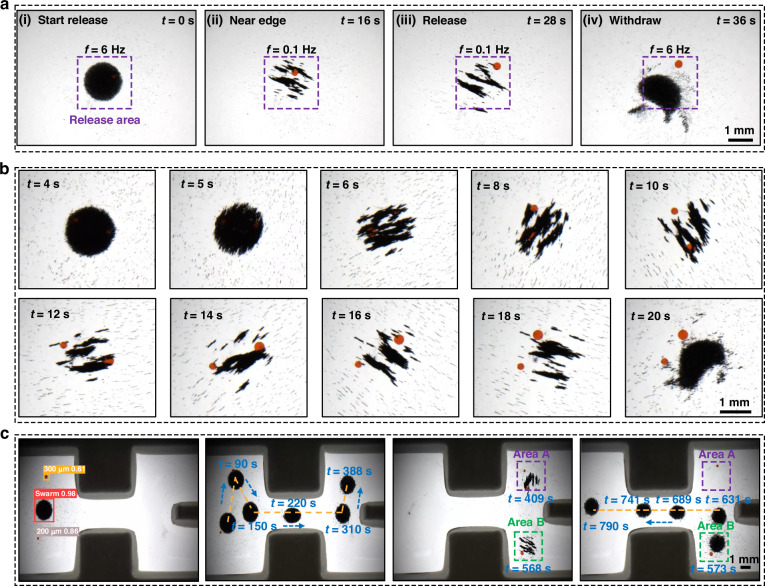
Controllable release of microcargo using the frequency-switching strategy. **a** Controllable release of a single PS by a microswarm based on frequency switching. **b** Release of 200 and 300 µm PSs via frequency-switching. **c** The microswarm captures 200 and 300 µm PSs and releases them to areas A and B, respectively

Figure 4a shows the controllable release mechanism based on frequency switching. When the microswarm carrying a PS with a diameter of 300 µm reaches the designated release area, f is first reduced to 0.1 Hz, which rapidly reorganizes the microchains into a long-chain structure. The thrust generated by the microchains gradually pushes the internal cargo toward the edge of the microswarm. After 12 s, the cargo detaches from the microswarm. Subsequently, f is increased to 6 Hz, causing the microchains to rapidly reconfigure into dense aggregates that move away from the release area (Supplementary Movie 4).

Furthermore, rod-shaped microchains fragmented during the manipulation of larger cargo (Fig. 4b). In contrast, when releasing smaller cargo, the microchain structure remained stable, and the lower hydrodynamic resistance of the smaller cargo enabled it to respond more readily to the thrust generated by the rotating rod-shaped microchain (Supplementary Movie 5). Therefore, in scenarios where a microswarm encapsulated two PSs, the smaller one was consistently released first. The 200 µm PS first reached the edge of the microswarm at 10 s. In contrast, when the microchains pushed the 300 µm PS, they were prone to breakage, which resulted in the 300 µm PS reaching the microswarm edge only at 18 s.

Leveraging this size-dependent release behavior, we conducted a multicargo delivery experiment in a microchannel (Fig. 4c and Supplementary Movie 6). After sequentially capturing PSs with diameters of 200 and 300 µm, the microswarm followed a predefined path through a 3mm-wide microchannel and delivered the cargos to distinct areas. Upon reaching Area A, the PS with a diameter of 200 µm was preferentially released. Subsequently, the microswarm reaggregated, moved to Area B, and released the PS with a diameter of 300 µm. After the release was completed, the empty microswarm returned to its initial position, enabling recovery of the magnetic particles. This process achieved programmed capture, delivery, and controllable release of multiple cargos, demonstrating the flexibility and scalability of the platform in multicargo and complex delivery scenarios.

### Bidirectional microcargo delivery in a fluidic flow environment

Fluid disturbances are inherent to in vivo delivery processes. The performance of the microswarm under physiologically relevant flow conditions was assessed via experiments conducted in a microfluidic channel with fetal bovine serum (FBS) as a simulated physiological fluid (Fig. S4). A stable laminar flow was established using a precision pump control system, with flow velocities of 100–500 µm/s to mimic the slow synovial fluid dynamics characteristic of the articular joint environment. The measured in vivo synovial fluid flow rates are 120–180 µm/s^50^, validating the physiological relevance of the tested velocity range.

**Fig. 5:**
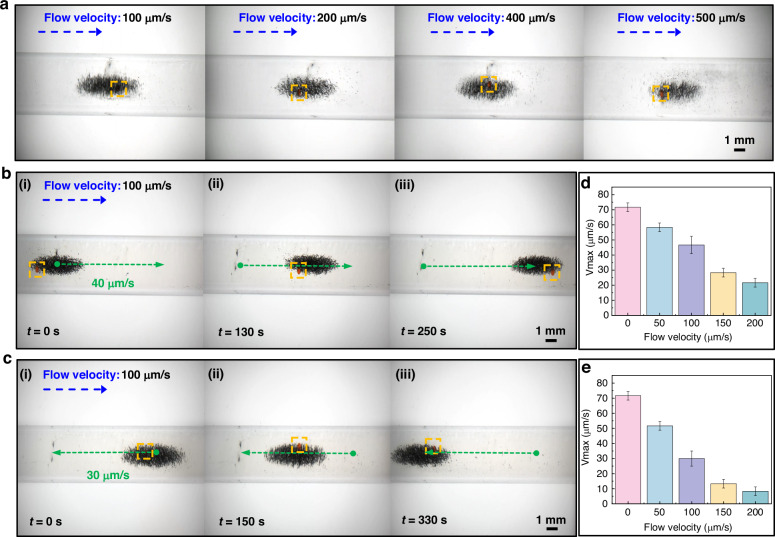
Delivery performance of magnetic microswarms under physiologically relevant flow conditions. **a** Stable encapsulation of a 300 µm PS by a microswarm immobilized in a microfluidic channel under laminar flow (100–500 µm/s). **b** Directional downstream transport of a 300 µm PS at a flow velocity of 100 µm/s (blue dashed line), achieved at a propulsion speed of 40 µm/s (green dashed line). **c** Upstream delivery of the cargo-carrying microswarm at 30 µm/s (green dashed line). **d** *V*_max_ of microswarms carrying a 300 µm PS under co-flow conditions (flow velocity range: 0–200 µm/s). **e** *V*_max_ of microswarms under counter-flow conditions (flow velocity range: 0–200 µm/s). The error bars represent the standard deviation from three independent experiments

Figure 5a shows that after the stable capture of a PS with a diameter of 300 µm, the magnetic microswarm is immobilized in a designated region to assess its cargo retention capability under dynamic flow conditions (Supplementary Movie 7). The microswarm completely encapsulates the cargo even at flow velocities up to 500 µm/s, and the absence of structural disintegration or active cargo detachment indicates its excellent structural stability. When the flow velocity exceeds 700 µm/s, the cargo gradually detaches from the microswarm (Fig. S5). By adjusting the particle dosage, magnetic field intensity and frequency, the resistance of the microswarm to hydrodynamic shear forces can be further improved, thus extending the manipulation range of the microswarm to harsher physiological fluid environments. Under co-flow and counter-flow conditions, the microswarm achieves stable, directional delivery of a 300 µm PS (Fig. 5b, c and Supplementary Movie 8). Guided by the magnetic tweezers, the microswarm maintains its structural integrity and effectively overcomes external fluidic disturbances to achieve sustained forward propulsion, demonstrating controllable motility and robust cargo retention under bidirectional flow conditions.

The *V*_max_ of the microswarm carrying a 300 µm PS under co-flow and counter-flow conditions, respectively, across a flow velocity range of 0–200 µm/s is presented in Fig. 5d, e. In the channel, the microswarm structure evolves into an ellipsoidal shape, and the maximum transportation speed at a flow velocity of 0 µm/s is ~70 µm/s, which is slightly lower than that in an open, unconfined environment. Furthermore, the experimental results show that *V*_max_ decreases with increasing flow velocity, indicating a resistive effect of the external flow field on microswarm motility. At a flow velocity of 200 µm/s, the *V*_max_ values are 20 and 10 µm/s in the co-flow and counter-flow directions, respectively. Despite the flow-induced suppression of motility, cargo encapsulation by the microswarm remained stable throughout the experiment, with no observed detachment or structural disintegration. These results confirm the feasibility and robustness of magnetically driven microswarms for targeted delivery under dynamic flow conditions and demonstrate their potential for functional applications in low-velocity physiological environments.

### Microcargo delivery in a complex microenvironment

**Fig. 6:**
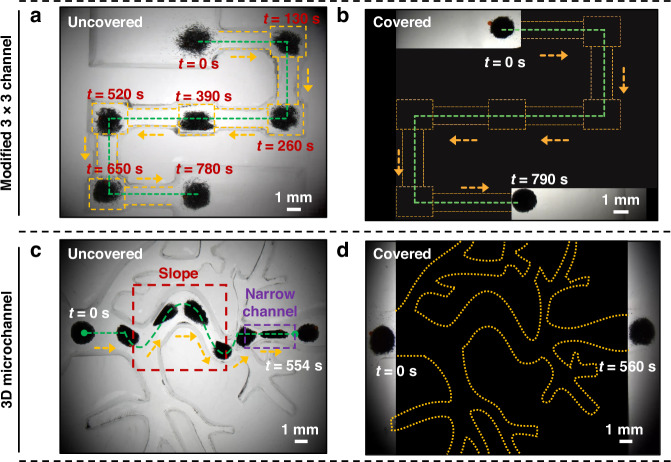
Navigation and delivery performance of a magnetic microswarm in complex microchannel structures. **a** Spatiotemporal images of the microswarm performing delivery of a 300 µm PS in a structurally modified 3 × 3 microchannel network. **b** The microswarm transports the 300 µm PS through the occluded structure to the target observation window, with channel sections covered except for the inlet and target areas. **c** The microswarm carries a 300 µm PS through the 3D microchannel. **d** The microswarm transports a 300 µm PS through the 3D microchannel featuring a visually occluded path

To evaluate the delivery capability of the developed system under physiologically relevant conditions, microchannels were designed to mimic the typical challenges encountered during microscale cargo transport in vivo (Fig. S6). Figure 6a and Supplementary Movie 9 show a magnetic microswarm performing navigated delivery within an open, structurally modified 3×3 microchannel. The morphological evolution of the microswarm during delivery can be observed in real time through the transparent microchannel. After stable capture of the PS with a diameter of 300 µm, the microswarm was guided by the magnetic tweezers to move directionally along a predefined trajectory at a speed of approximately 45 µm/s. When navigating a turn of 90°, the microswarm smoothly completes the direction change.

Navigation of the microswarm was fully controlled by the magnetic tweezers, enabling the cargo-carrying microswarm to navigate without real-time visual feedback. As shown in Fig. 6b and Supplementary Movie 9, all sections of the microchannel, except for the inlet and target observation windows, were fully covered, thereby obstructing the visual monitoring of the intermediate path. The microswarm successfully transported the PS from the entrance through the occluded structure and accurately reached the target observation window.

Furthermore, the cargo delivery through a three-dimensional (3D) microchannel was successfully demonstrated. This architecture incorporated bifurcated pathways, slope channels, and narrow channels. After the stable capture of the 300 µm PS, the microswarm was sequentially guided through these structural elements (Fig. 6c and Supplementary Movie 10). In open regions, the microswarm maintained a quasi-circular or ellipsoidal configuration, maintaining a certain distance from the channel walls. When traversing inclined channels with a slope of ±15°, the microswarm deformed into an elliptical shape but continued to exhibit excellent motion stability and payload retention capability. Upon entering a narrow channel with a width of 1 mm, it underwent reversible compression, conforming closely to the channel sidewalls while maintaining structural integrity and sustaining forward propulsion. We further performed experiments in which the microswarm passed through channels of varying widths. Although the maximum transportation speed decreased after morphological deformation, the microswarm still maintained stable cargo capture and reliable delivery throughout the process (Fig. S7). The experimental results demonstrate that magnetic microswarms exhibit excellent path adaptability and structural stability.

Autonomous navigation of the microswarm was successfully achieved in the 3D microchannel even with visually occluded intermediate paths (Fig. 6d and Supplementary Movie 10). During the delivery process, the imaging module was used to record the motion trajectory and verify the final delivery state, but did not provide real-time visual feedback for closed-loop control. The microswarm navigated along a predefined route, which enabled reliable cargo delivery even under visually occluded conditions and expanded the practical application scope of the system to opaque or confined biological environments.

### Microcargo delivery on the bone fragment model

**Fig. 7:**
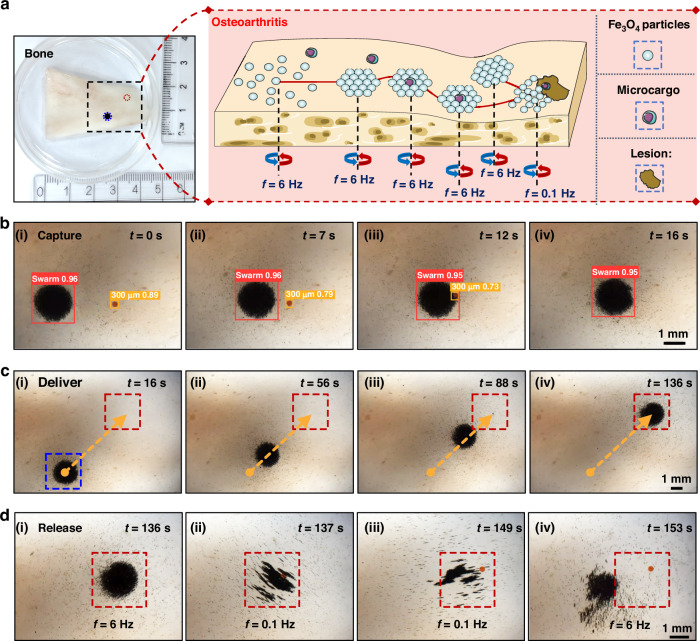
Delivery performance of magnetic microswarm on a bone surface in vitro. **a** Structure of the bone model and its conceptual diagram for application in osteoarthritis treatment. **b** The microswarm actively searches and stably encapsulates the PS with a diameter of 300 µm within 16 s, even under strong optical scattering and shadowing from the bone surface. **c** The microswarm transported the cargo along a predefined trajectory to the target region (red dashed box) at a speed of ~50 µm/s. **d** Frequency-switching-triggered cargo release and microswarm recovery on the bone model surface

Although stem cell therapy holds great promise for osteoarthritis treatment, its clinical translation is hindered by the lack of delivery strategies that are both efficient and precisely targeted^51^. In this study, an ex vivo bone fragment model was established using porcine femur tissue to realistically replicate the complexity of the in vivo environment (Fig. 7a). This model replicated the surface topography, mechanical hardness, and irregular microscale texture of human bone tissue and preserved the characteristic local concavities of native bone. In this model environment, the magnetic tweezers system integrated with the deep visual recognition successfully achieved real-time localization and dynamic tracking of the microswarm and PS (Supplementary Movie 11). Even under the interference of strong scattering and local shadows on the bone surface, the system accurately identified the target information and maintained stable operation. Within 16 s, the microswarm completed active searching and stable encapsulation of the 300 µm PS, showing excellent response speed and manipulation precision (Fig. 7b).

After successful capture, the microswarm stably advanced toward the center of the target region along a predefined trajectory at a constant speed of ~50 µm/s (Fig. 7c). Throughout the delivery process, the microswarm maintained a dense aggregate morphology and did not disintegrate owing to bone surface roughness or local flow field disturbances. This characteristic facilitates reliable drug delivery at complex biological interfaces. Upon reaching a predetermined position, the release mechanism was triggered by adjusting the magnetic field frequency. The PS moved to the edge of the microswarm after 12 s (Fig. 7d). Subsequently, the microswarm rapidly reconfigured into a compact state and withdrew directionally when the high-frequency magnetic field was restored.

These results demonstrate that the system can efficiently and precisely deliver nonmagnetic functional microcargos in a simulated osteoarticular environment. Meanwhile, the effectiveness of the proposed release mechanism was further validated in the environment, with the microswarm successfully releasing the cargo and exiting the release zone. During locomotion of the microswarm on the bone surface, minor shedding of Fe_3_O_4_ nanoparticles may occur due to physical interactions. The shed nanoparticles only account for a small fraction of the total swarm mass, and most nanoparticles can be retrieved after cargo delivery. Thus, the potential residuals pose a significantly lower safety risk compared to conventional magnetically labeled targets. This approach provides a reliable proof of concept for osteoarthritis treatment, bone defect repair, and related musculoskeletal disorders.

### Automated delivery of functional cell spheroids

**Fig. 8:**
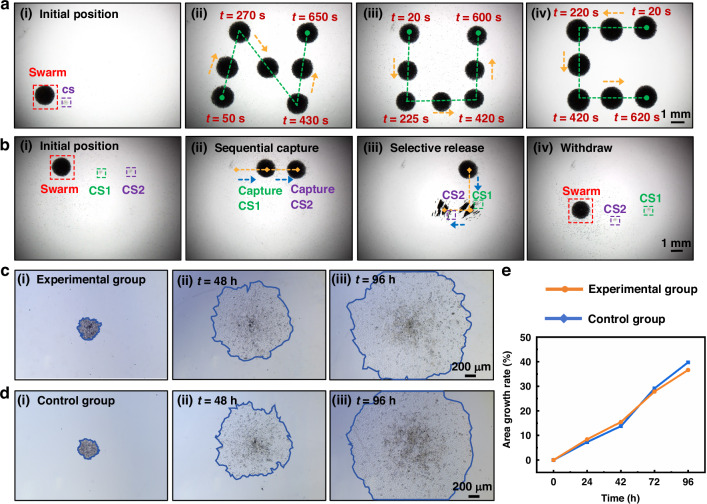
Delivery performance of a magnetic microswarm of CSs. **a** The microswarm captured a CS with a diameter of 300 µm and stably delivered it along predefined “N”, “U”, and “C” shaped trajectories. **b** The microswarm captured CS1 (300 µm, green dashed box) and CS2 (400 µm, purple dashed box), and released them at different sites. **c** Morphological evolution of CSs in the experimental group (subjected to microswarm capture-delivery-release) after 0, 48, and 96 h of culture. **d** Morphological evolution of CSs in the control group (no manipulation) after 0, 48, and 96 h of culture. The blue solid line denotes the expanded area of the CSs. **e** Proliferation curve of CSs (experimental and control groups)

To evaluate the biocompatibility of magnetic microswarm-mediated delivery, HeLa cell spheroids (CSs) with diameters of 300–400 µm were cultured as nonmagnetic microcargo (Fig. S8). The microswarm successfully captured a single CS with a diameter of 300 µm and stably delivered it along the predefined trajectories, including “N,” “U,” and “C” shapes (Fig. 8a and Supplementary Movie 12).

To further verify the effectiveness of the proposed frequency-switching release strategy, a CSs delivery and stepwise release experiment was conducted following the proposed approach (Fig. 8b and Supplementary Movie 13). The microswarm sequentially captured two CSs of different sizes: CS1 (~300 µm) and CS2 (~400 µm), and co-delivered them to the target region. After triggering the release mechanism, the smaller spheroid (CS1) was released at the first target area, whereas the larger spheroid remained encapsulated within the microswarm. Subsequently, CS2 was transported to the next location and released. Finally, the microswarm returned to the origin.

Further, experimental and control groups were established for comparative analysis to evaluate the impact of the delivery process on the physiological state of CSs. The experimental group consisted of CSs that underwent microswarm capture, directional delivery, and release, whereas the control group included size-matched isogenic CSs without any manipulation. Both groups of samples were cultured synchronously under the same culture conditions, and their morphological evolution and proliferation behaviors were periodically observed. Spheroids in both groups gradually adhered to the substrate and initiated spreading, indicating the preservation of basic physiological functions (Fig. 8c, d). The outgrowth area of cell spheroids was quantitatively analyzed using ImageJ software at 24 h intervals over a total culture period of 96 h. The area growth rate was calculated using the initial spheroid area at 0 h for normalization, according to the following formula:

1 $${\rm{Area\; Growth\; Rate}}( \% )=\frac{{{\rm{A}}}_{{\rm{t}}}-{{\rm{A}}}_{0}}{{{\rm{A}}}_{0}}\times 100 \%$$

where A_0_ represents the initial attachment area (0.13 mm^2^ for both groups), and A_t_ denotes the outgrowth area after t hours of culture.

The area growth rates of the two groups are presented in Fig. 8e. Both groups exhibited continuous outgrowth and peripheral cell migration, with highly similar proliferative kinetics and no significant growth delay. After 48 h of culture, the cell area growth rates of the experimental and control groups were 15.4 and 13.7%, respectively, while they reached 36.6 and 39.7%, respectively, after 96 h of culture. These results indicate that the magnetic microswarm-mediated delivery process did not induce detectable adverse effects on the structural integrity, adhesion capacity, or long-term proliferative potential of the CSs. The excellent biocompatibility and operational safety demonstrate the potential of this approach for applications in in vivo tissue engineering and cell-based therapies.

## Conclusions

This paper describes a label-free strategy for manipulating and delivering nonmagnetic microcargo using a magnetic tweezers system. A reconfigurable microswarm with tunable morphologies was formed via the self-assembly of Fe_3_O_4_ nanoparticles, enabling efficient cargo capture, stable transport, and frequency-switching-based release. High-frequency magnetic fields enabled compact swarms for secure transport, whereas low-frequency magnetic fields facilitate controllable release. The integration of the YOLOv5 detection algorithm further enhanced automation and enabled the sequential capture of multiple microcargos. By tuning the magnetic field parameters, the disturbance rejection capability of the cargo-carrying microswarm was further enhanced. The cargo-carrying microswarm demonstrated stable navigation in a dynamic fluidic environment, 3D microchannels, and a bone fragment model, confirming its robust adaptability to complex fluidic environments. In addition, CSs delivered via the microswarm exhibited proliferation comparable to that of the controls, demonstrating the excellent biocompatibility of the microswarm-based delivery mechanism. Moreover, the microswarm enabled multipoint, controllable release of microcargos via magnetic frequency-switching mechanism, significantly broadening the scope of microrobotic applications. However, several current challenges remain, including constraints on the types and loading capacities of cargos, as well as insufficient in vivo biocompatibility validation. Future research will focus on integrating drug and stem cell delivery to develop precision therapeutic systems with strong clinical potential, particularly in the treatment of osteoarthritis and vascular-related diseases. Further optimization of material design and control strategies will be essential to promote the practical clinical translation of such microrobotic systems.

## Materials and methods

### Experimental setup

The imaging device consisted of a CMOS camera (Hikvision, MV-CA050-12UC) and a microscopic lens mounted vertically above the base platform to capture high-resolution images of the operation area. The host computer employed a custom graphical user interface developed using the Qt Designer, which supported real-time image processing and motion path planning. The base plate incorporated a light-diffusing plate embedded in a light-emitting diode array to provide uniform illumination. Magnetic actuation was achieved using two permanent magnets mounted on a rotating disk, driven by a motor with a rotation frequency tunable from 0 to 10 Hz. Grade N52 magnets that measured 10 mm × 10 mm × 10 mm were used. The rotor was mounted on a translation stage to enable precise field control. The system dynamically regulated microswarm morphology and enabled directional transport by synchronizing the rotation frequency and stage position.

### Magnetic field simulation

The magnetic field distribution was simulated and analyzed using COMSOL Multiphysics software to clarify its spatial characteristics. A simulation model was established with the geometric center of the two permanent magnets as the coordinate origin. The permanent magnets were represented as cubic geometries with a side length of 10 mm, and their magnetization directions were set to be opposite. The material of the permanent magnets was defined as N52 (sintered NdFeB) with a residual magnetic flux density of 1.44 T. To ensure the accuracy and reliability of the simulation results, a sufficiently large rectangular air domain was constructed to completely enclose the magnet structure, eliminating boundary effects on the magnetic field distribution.

### Self-assembly of microswarms

Reconfigurable microswarms were formed using superparamagnetic Fe_3_O_4_ nanoparticles (0.5–0.6 µm). The Fe_3_O_4_ nanoparticles were purchased from the Tianjin Baseline ChromTech Research Center (Tianjin, China). The nanoparticles were dispersed in phosphate-buffered saline (PBS, 10010023, Gibco) to form a homogeneous suspension at a concentration of 0.5% (w/v). The dynamic self-assembly of magnetic nanoparticles into reconfigurable microswarms was induced by an external rotating magnetic field.

### Fluid simulation

The flow field induced by the microswarm was simulated using COMSOL Multiphysics software. Simulation models were constructed based on a chain length of 45 µm at 10 Hz. The microchain structures were simplified as rectangular geometries with lengths of 100, 80, and 45 µm, with spacings twice the lengths. These configurations corresponded to the typical microswarm configurations observed experimentally under f = 3 Hz, f = 6 Hz, and f = 10 Hz, respectively. All rectangular simplifications were set to rotate about their own central axes at the corresponding specified frequency. A large square was employed as the fluid computational domain. The working fluid was set as water, with a dynamic viscosity of 0.001 Pa•s.

### Production of model channels

Microchannels were fabricated via 3D printing, using polylactic acid and transparent photosensitive resin as the printing materials. Fluid dynamics experiments were conducted in a circular glass tube with an inner diameter and length of 2.2 and 50 mm, respectively, and a controllable flow environment was provided by a syringe pump (TS-1B, Longer). The bone fragment model was fabricated using ex vivo porcine bone sections. The porcine bone was cut into thin slices that were approximately 3 mm thick. After surface cleaning, the slices were used to simulate the environment of the bone defect.

### Cell spheroids (CSs) production

CSs were prepared in U-bottomed 96-well plates. HeLa cells were cultured in Dulbecco’s Modified Eagle Medium (DMEM) (11885084, Gibco) supplemented with 10% fetal bovine serum (10270106, Gibco), penicillin (100 U mL^−1^), and streptomycin (100 U mL^−1^, 15140122, Gibco). Cells were rinsed with PBS (10010023, Gibco), digested with 0.25% trypsin (25200072, Gibco), neutralized with complete medium after digestion, and resuspended to 3–5 × 10^4^ cells/mL. About 50 μL of cell suspension was seeded per well and cultured at 37 °C with a 5% CO_2_ atmosphere. After 48 h, cells formed spheroids with diameters of 300–400 μm. Images were acquired using an inverted fluorescence microscope (WMF-3680, Wumo).

### Calculation of the cell spheroid area

Cell spheroid images were quantitatively analyzed using ImageJ software. Images were converted to 8-bit grayscale to enhance contrast. After calibrating the image scale, the spheroid boundary was manually outlined to select the target region, with the software automatically calculating the selected area. To minimize measurement errors, each spheroid was measured three times independently, and the average value was used as the final area.

## Supplementary information

Supplementary Information

Microswarm navigation along a predefined triangular trajectory

Autonomous capture of a single PS by a microswarm with YOLOv5s-based visual feedback

The microswarm autonomously captures PSs sequentially in ascending order of size

Controllable release of a single PS based on frequency-switching strategy

Controllable release of 200 µm and 300 µm PS based on frequency-switching strategy

Sequential capture and delivery of 200µm and 300µm PSs by a magnetic microswarm

Stable encapsulation of a 300 µm PS by the microswarm under fluidic environment

Downstream and upstream delivery of the cargo-carrying microswarm

Autonomous navigation of the microswarm carrying a PS in a structurally modified 3 × 3 microchannel

Autonomous navigation of the microswarm carrying a PS through a 3D microchannel

The microswarm delivers a 300 µm PS on the bone surface

The microswarm delivers a 300-µm cell spheroid

The microswarm delivers cell spheroids with diameters of 300 and 400 µm

## Data Availability

All data supporting the findings of this study are available within this paper and its Supplementary Information file. All other relevant data were available from the corresponding author upon request.
