# Supplementary material for: Label-free microcargo delivery and controllable release in complex fluidic environments based on autonomous magnetic microswarm
Source: Microsyst Nanoeng. 2026 Jul 24;12:276. doi: 10.1038/s41378-026-01392-0 (PMC13400644; doi:10.1038/s41378-026-01392-0)
Supplement: Supplementary file 1 — Supplementary Information [file 41378_2026_1392_MOESM1_ESM.docx]

Supplementary Information for

**Label-Free Microcargo Delivery and Controllable Release in Complex Fluidic Environments Based on Autonomous Magnetic Microswarm**

Zhixin Wu^1,2,†^, Liushuai Zheng^3,†^, Lei Fan^1,2,^*, Tanyong Wei^4^, Jinyang Gao^1,2^, Xue Qi^1,2^, Lei Zhang^1,2^, Huanting Shi^1,2^, Bingdan Wang^1,2^, Renkai Guo^1,2^, Hao Yan^5,^*, Qiulin Tan^1,2,^*

^1^ State key Laboratory of Extreme Environment Optoelectronic Dynamic Measurement Technology and Instrument, North University of China, Taiyuan 030051, China

^2^ Key Laboratory of Micro/nano Devices and Systems, Ministry of Education, North University of China, Taiyuan 030051, China

^3^ School of Mechanical Engineering, Suzhou University of Science and Technology, Suzhou, 215000, China

^4^ School of Mechanical Engineering, Hubei University of Technology, Wuhan 430068, China

^5^ Department of Biliary and Pancreatic Surgery, First Hospital of Shanxi Medical University, Taiyuan 030001, China

^†^ These authors contributed equally: Zhixin Wu, Liushuai Zheng

*Corresponding authors.E-mail: [20230054@nuc.edu.cn](mailto:20230054@nuc.edu.cn) (Lei Fan); [yanhao198603@163.com](mailto:yanhao198603@163.com) (Hao Yan); [tanqiulin@nuc.edu.cn](mailto:tanqiulin@nuc.edu.cn) (Qiulin Tan)

**1. Supplementary Movie Description**

**Supplementary Movie 1.** Microswarm navigation along a predefined triangular trajectory

**Supplementary Movie 2.** Autonomous capture of a single PS by a microswarm with YOLOv5s-based visual feedback

**Supplementary Movie 3.** The microswarm autonomously captures PSs sequentially in ascending order of size

**Supplementary Movie 4.** Controllable release of a single PS based on frequency-switching strategy

**Supplementary Movie 5.** Controllable release of 200 µm and 300 µm PS based on frequency-switching strategy

**Supplementary Movie 6.** Sequential capture and delivery of 200 µm and 300 µm PSs by a magnetic microswarm

**Supplementary Movie 7.** Stable encapsulation of a 300 µm PS by the microswarm under fluidic environment

**Supplementary Movie 8.** Downstream and upstream delivery of the cargo-carrying microswarm

**Supplementary Movie 9.** Autonomous navigation of the microswarm carrying a PS in a structurally modified 3×3 microchannel

**Supplementary Movie 10.** Autonomous navigation of the microswarm carrying a PS through a 3D microchannel

**Supplementary Movie 11.** The microswarm delivers a 300 µm PS on the bone surface

**Supplementary Movie 12.** The microswarm delivers a 300 µm cell spheroid

**Supplementary Movie 13.** The microswarm delivers cell spheroids with diameters of 300µm and 400µm

**2. Supplementary Figures**


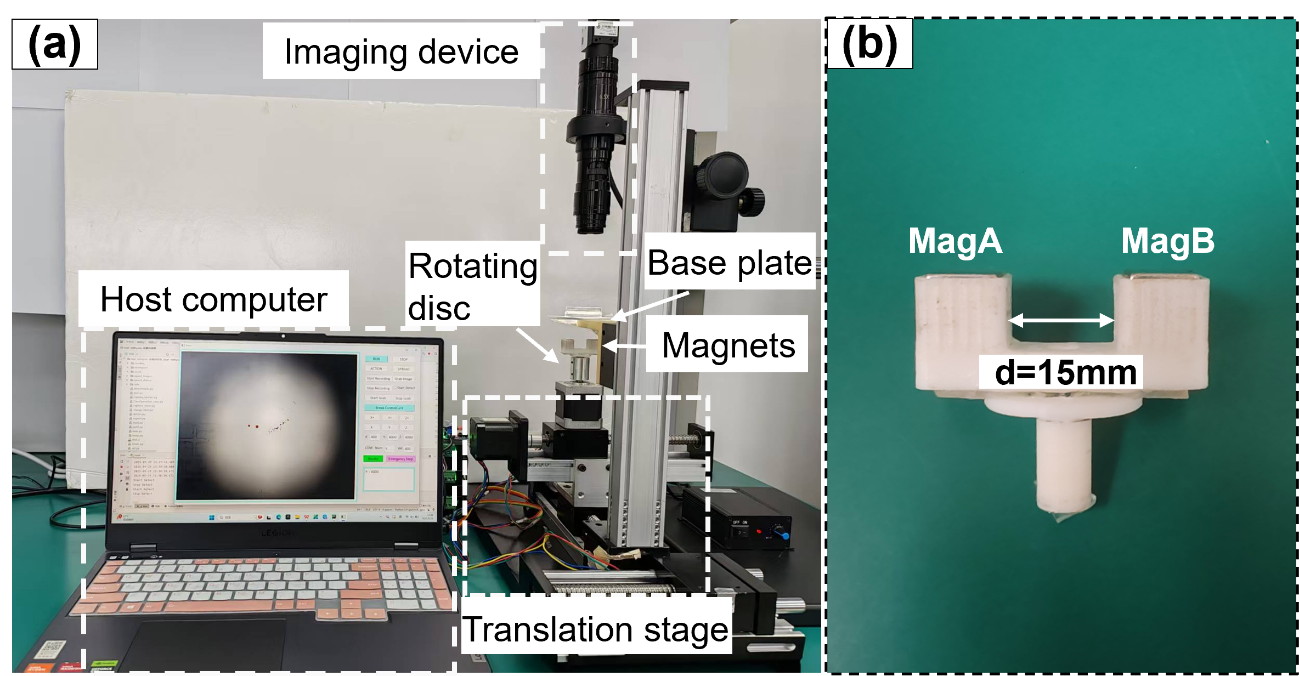


**Fig. S1. The magnetic tweezers system for this study. (a)** Schematic illustration of the magnetic tweezers system. The magnetic tweezer system comprised an imaging device, a host computer, a base plate, permanent magnets, a rotating disk, and a translation stage. **(b)** A rotating disk drives two permanent magnets to generate a dynamic magnetic field, with the distance between the magnets fixed at 15 mm.


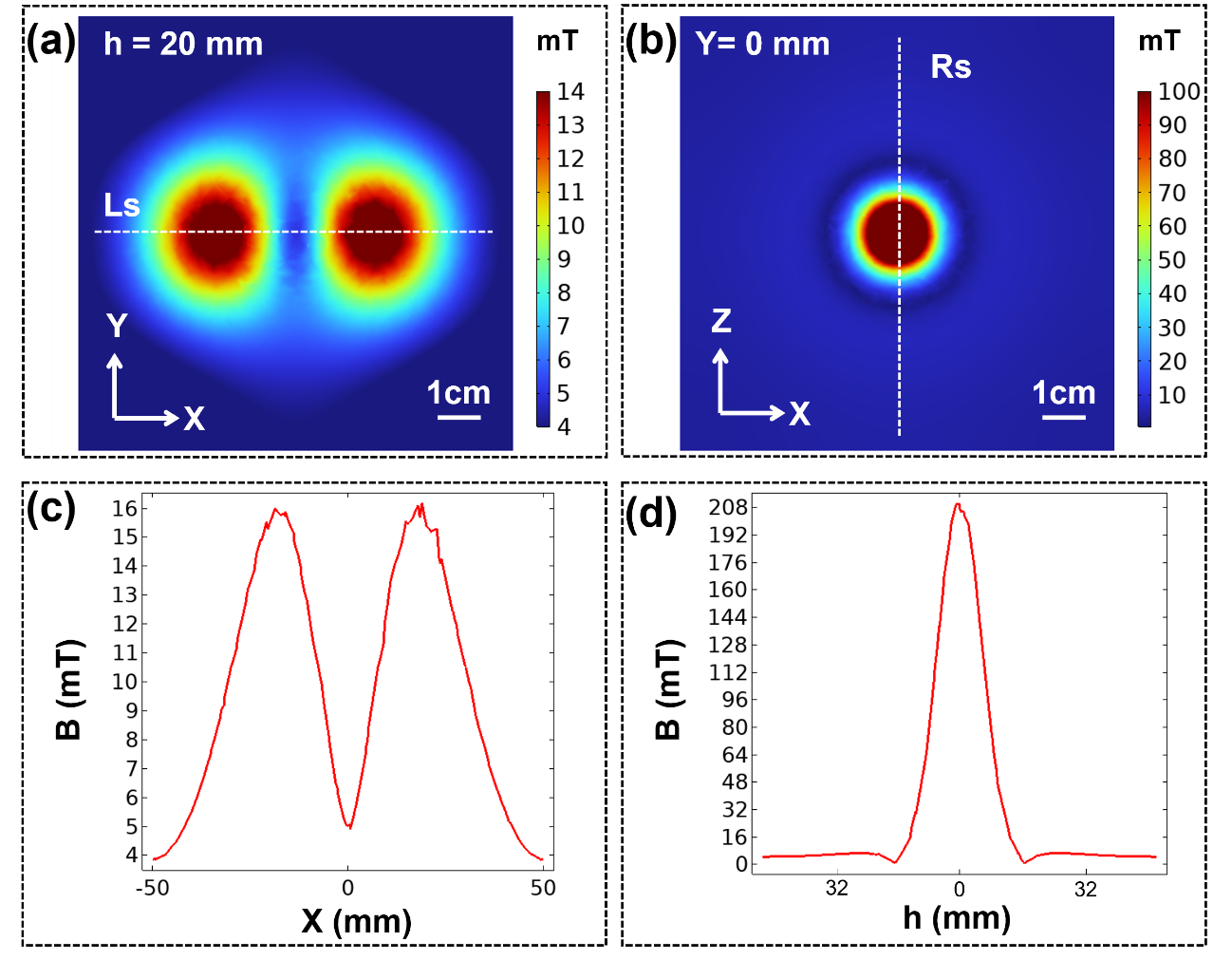


**Fig. S2. Numerical simulation of the magnetic field distribution. (a)** The magnetic field distribution in the XY plane at a height h = 20 mm. **(b)** The magnetic field distribution in the XZ plane. **(c)** Variation of magnetic induction intensity (B) along Ls at h = 20 mm. The field exhibits a characteristic "V" shape, reaching its minimum at the center of the working region. **(d)** Variation of magnetic induction intensity (B) along Rs. The field intensity at the center decreases with increasing h, followed by a slight increase.


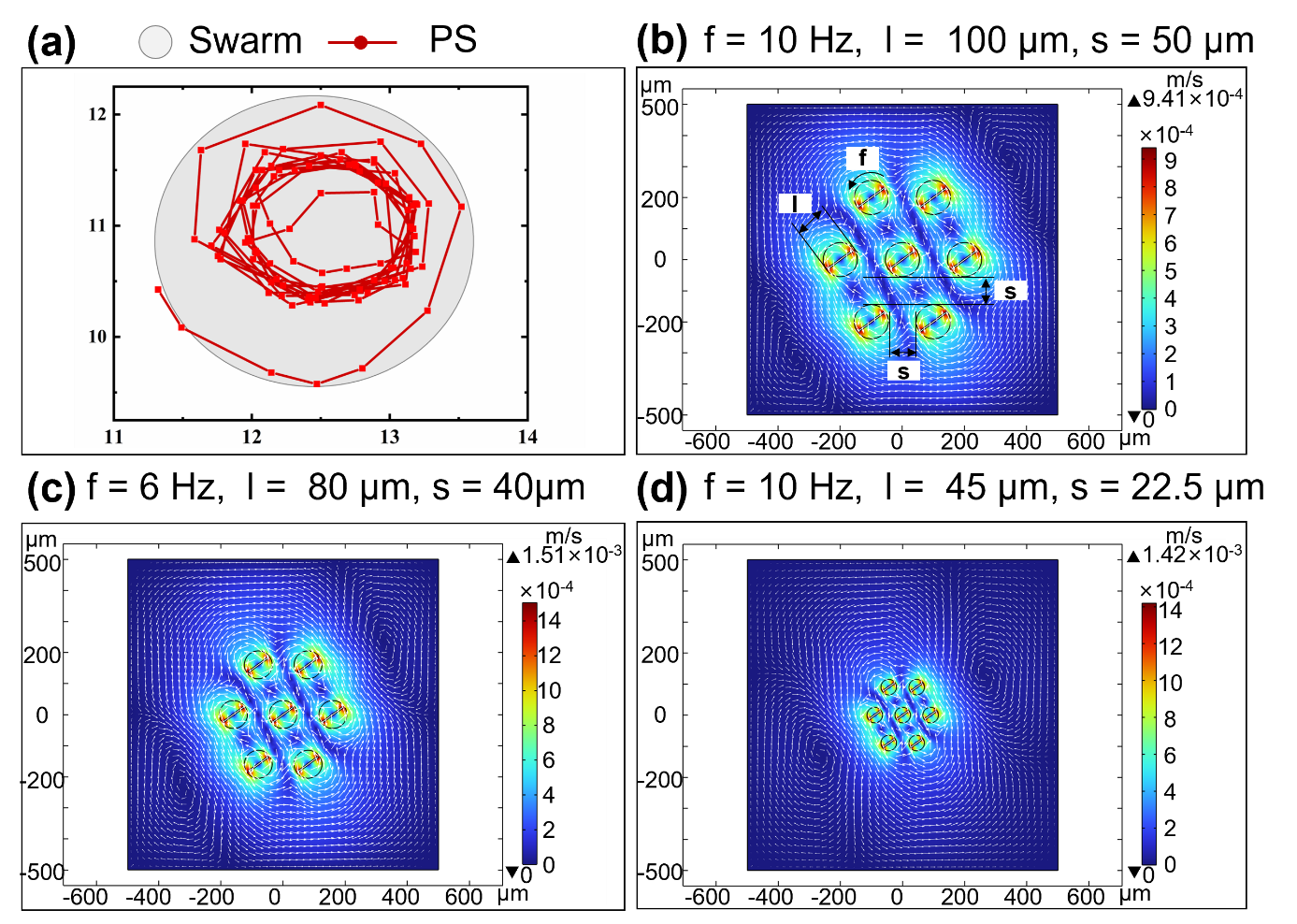


**Fig. S3. Motion of PS within the microswarm and representative images of microswarm configurations and simulated flow fields.** **(a)** A 300 μm PS was captured by the microswarm, and its trajectory was recorded. The PS moved toward the swarm center along a spiral trajectory. **(b-d)** The simulation results showed that the rotational motion of the internal microchains generated a vortex-like flow field. At f = 3 Hz, the over-elongated microchains generated low fluid velocity, with a maximum flow velocity (V_f_) of 9.41×10^-4^ m/s. Increasing f to 6 Hz led to shorter microchains and a higher fluid velocity, with V_f_ reaching 1.51×10^-3^ m/s. However, at f = 10 Hz, the excessively short microchains formed a dense aggregate structure. This aggregation restricted internal fluid motion, resulting in a decrease in V_f_ to 1.42×10^-3^ m/s. The reduction in Vf diminished the pressure gradient within the microswarm, ultimately impairing the efficiency of cargo delivery mediated by the microswarm.


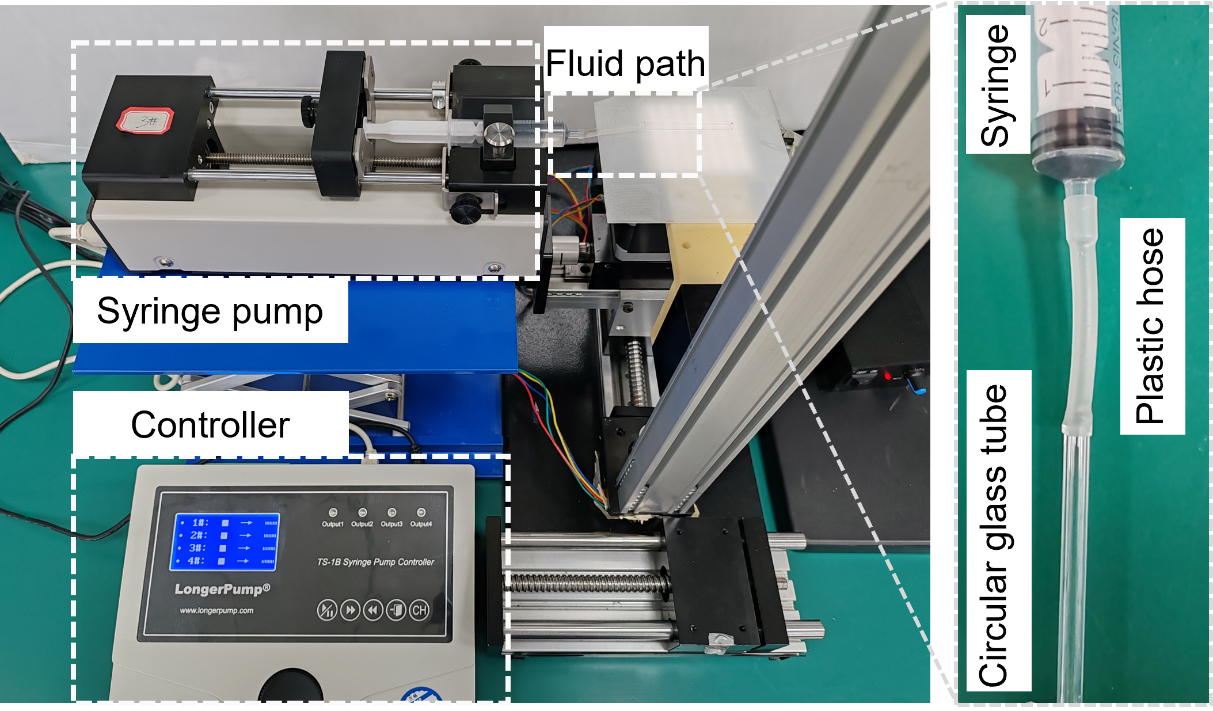


**Fig. S4.** **The fluid dynamics system used for this study.** A controllable flow environment was provided by a syringe pump and controller. Experiments were conducted in a circular glass tube with an inner diameter of 2.2 mm and a length of 50 mm. A plastic hose connected the syringe to the glass tube, allowing fluids to be pumped through the system via the syringe pump.


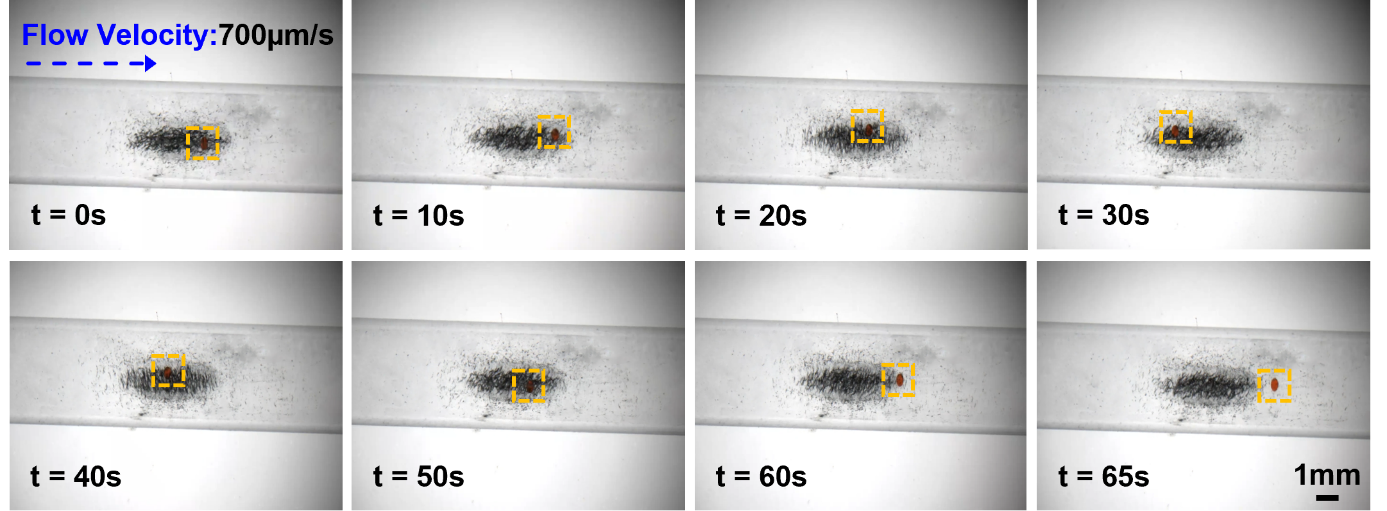


**Fig. S5. Cargo detachment from the microswarm at a flow velocity of 700 µm/s.**


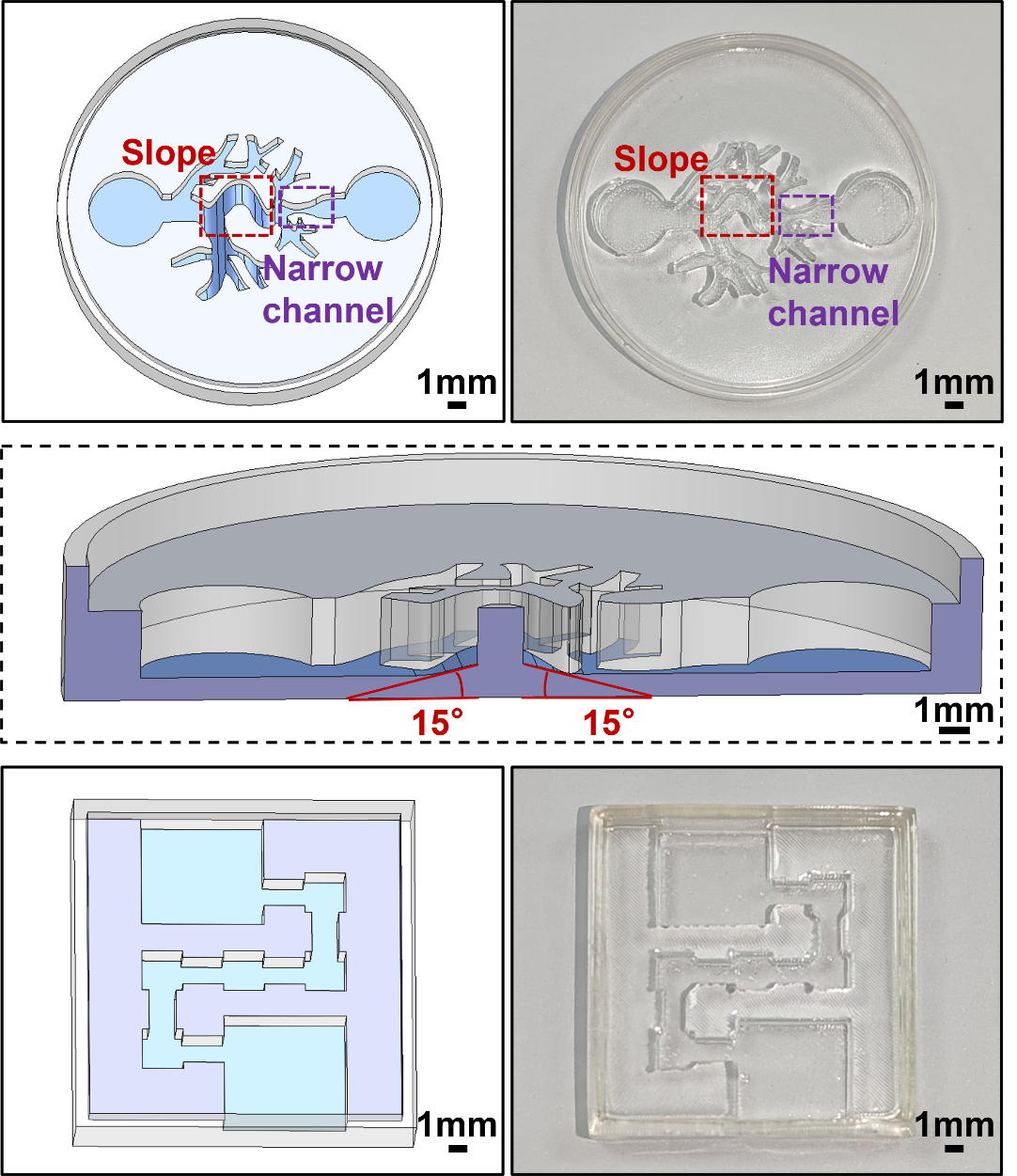


**Fig. S6. Microchannel models and physical prototypes.** After constructing the 3D channel model, the channel was printed using transparent resin to obtain the physical prototype. The transparent structure of the printed channel enabled real-time observation of microswarm motion during experiments.


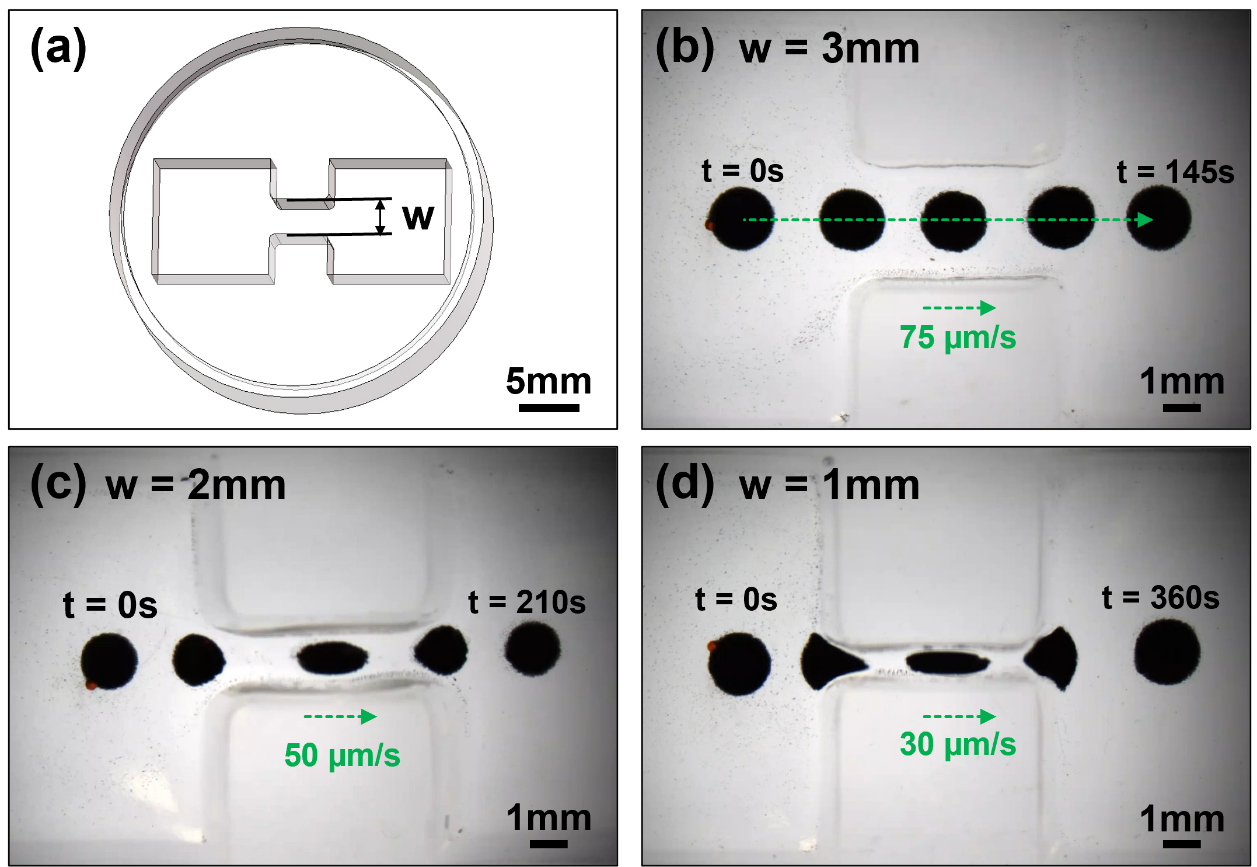


**Fig. S7. Performance of microswarm cargo transport through microchannels with varying widths. (a)** Schematic illustration of the microchannel model, where w denotes the channel width. **(b)** In the 3 mm wide channel, the microswarm successfully transports 300 µm PS at a velocity of 75 µm/s. **(c)** In the 2 mm wide channel, the microswarm maintains cargo capture while navigating at 50 µm/s. **(d)** In the 1 mm wide channel, the microswarm undergoes morphological deformation but achieves reliable delivery of the cargo at 30 µm/s, demonstrating stable functionality under physical constraints.


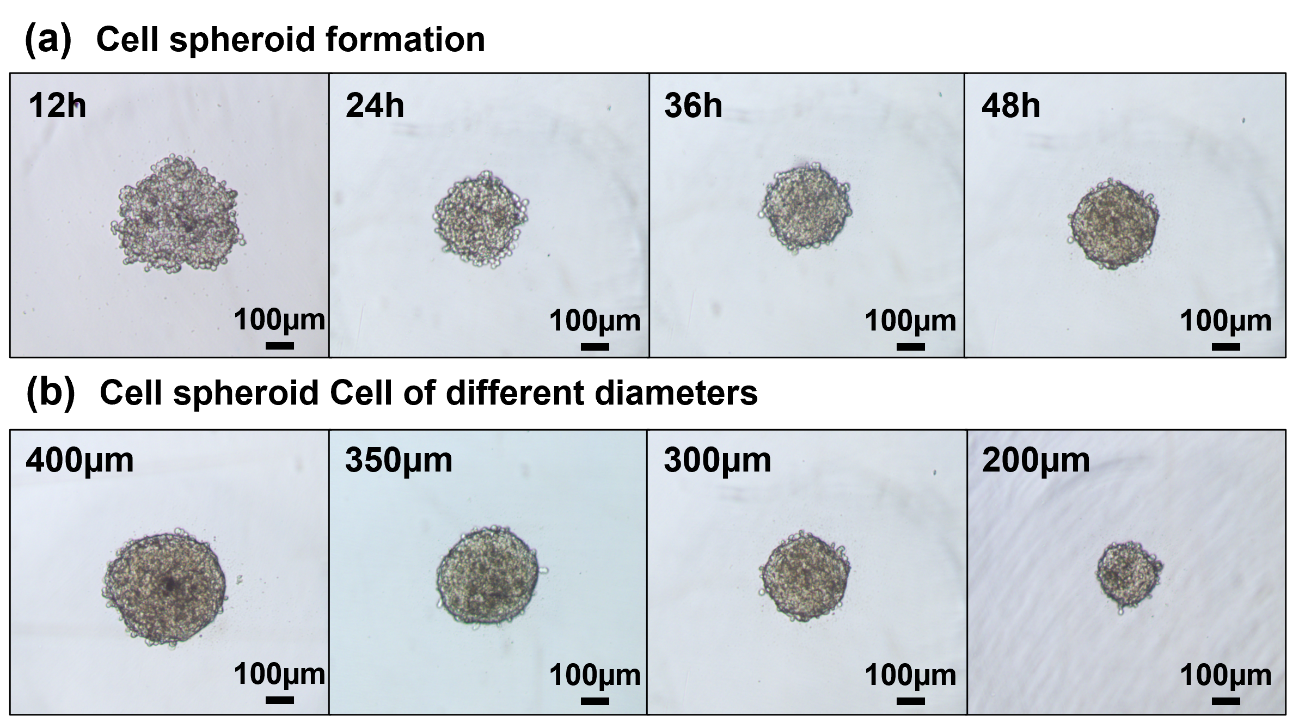


**Fig. S8. Formation of cell spheroids and images of cell spheroids with different diameters. (a)** Cells were digested to prepare a cell suspension. The concentration of the cell suspension was adjusted to 3×10^4^ cells/mL, and 50 μL of the cell suspension was seeded into each well. After culturing on low-adhesion surfaces for 48 hours, the cells self-aggregated to form cell spheroids with a diameter of 300 μm. **(b)**The diameter of cell spheroids could be controlled by adjusting the concentration of the cell suspension. When the concentration of the cell suspension was adjusted to 5×10^4^ cells/mL, cell spheroids with a diameter of 400 μm were obtained.
